# Clot-regression effects of rivaroxaban in venous thromboembolism treatment in cancer patients—a prospective interventional study

**DOI:** 10.1038/s41598-022-26150-w

**Published:** 2022-12-13

**Authors:** Shigeki Takai, Naohiko Nakanishi, Isao Yokota, Kojiro Imai, Ayumu Yamada, Takanori Kawasaki, Takeru Kasahara, Takashi Okada, Takahisa Sawada, Satoaki Matoba

**Affiliations:** 1 Department of Cardiovascular Medicine, Graduate School of Medical Science, Kyoto Prefectural University of Medicine, 465 Kajii-cho Kawaramachi-Hirokoji, Kamigyo-ward, Kyoto, 602-8566 Japan; 2 Department of Biostatistics, Graduate School of Medicine, Hokkaido University, Hokkaido, Japan; 3 Department for Medical Innovation and Translational Medical Science, Graduate School of Medical Science, Kyoto Prefectural University of Medicine, Kyoto, Japan; 4 The Clinical and Translational Research Center, Kyoto Prefectural University of Medicine, Kyoto, Japan; 5 Department of Cardiovascular Medicine, North Medical Center Kyoto Prefectural University of Medicine, Kyoto, Japan; 6 Department of Cardiovascular Medicine, Kyoto City Hospital, Kyoto, Japan; 7 Department of Cardiovascular Medicine, Japanese Red Cross Kyoto Daiichi Hospital, Kyoto, Japan; 8 Department of Cardiovascular Medicine, Japanese Red Cross Kyoto Daini Hospital, Kyoto, Japan

**Keywords:** Thromboembolism, Thrombosis

## Abstract

Rivaroxaban, a direct oral anticoagulant, is effective against venous thromboembolism (VTE) recurrence without increasing the risk of major bleeding in patients with cancer-associated venous thromboembolism (CAT). However, its clot regression effects are poorly understood. This single-arm, prospective interventional study aimed to investigate the clot regression effects of rivaroxaban in 40 CAT patients, through a contrast-enhanced computed tomography at baseline, 3 weeks, and 3 months of rivaroxaban treatment. The primary endpoint was the clot-regression ratio calculated from the thrombus volumes at 3 weeks and 3 months. Compared with baseline, the total clot volume was significantly reduced at both 3 weeks and 3 months after initiation (*p* < 0.01). The clot-regression rates were statistically significant with 83.1% (95% confidence interval [CI], 73.8–92.3%) at 3 weeks and 98.7% (95% CI, 97.1–100.2%) at 3 months, with complete resolution in 36.1% and 80.8% of patients at 3 weeks and 3 months, respectively. One patient had recurrent VTE after dose reduction, and seven had non-fatal major bleeding. Therefore, rivaroxaban had a sufficient clot-regression effect against CAT with caution of bleeding complication.

## Introduction

Cancer is a major risk factor for venous thromboembolism (VTE)^1,2^. Advances in cancer treatment have fortunately meant that the number of patients surviving after diagnosis is steadily increasing; however, this has concurrently been met with cancer-associated venous thromboembolism (CAT) emerging as a clinical issue. VTE increases the risk of death, which can directly affect cancer patient prognosis^3,4^. Conversely, active cancer represents a risk factor for both VTE recurrence and severe bleeding^5,6^, stressing the need for careful consideration of the type of anticoagulant used for therapy. Previous studies have reported that low-molecular-weight heparin (LMWH) reduces VTE recurrence without increasing hemorrhage risk in cancer patients as compared to vitamin K antagonists^7–9^, resulting in recommending LMWH as monotherapy for VTE in cancer patients^10,11^.

In recent years, direct oral anticoagulants (DOACs) have been employed as an alternative treatment for VTE in cancer patients. In this regard, DOACs were non-inferior to LMWH in treating VTE recurrence without increasing significant bleeding^12,13^. While rivaroxaban has been associated with a relatively low VTE recurrence rate and equivalent major bleeding, its clinically relevant non-major bleeding rate was higher than that of LMWH^14,15^. Based on these studies, DOACs have been recommended as an LMWH-alternative anticoagulant therapy for patients with CAT^10,11,16^. While the efficacy and safety of DOACs for VTE recurrence in CAT patients have been demonstrated, their clot regression effects in CAT patients with a hypercoagulation state remain unclear.

Incomplete clot resolution and residual thrombus lead to an increased risk of deep vein thrombosis (DVT) recurrence. DVT recurrence not only causes symptom reappearance but also poses a risk for post-thrombotic syndrome (PTS) development^17,18^. PTS is treatment-refractory and significantly reduces patients’ quality of life, highlighting the importance of PTS prevention. Similarly, the incomplete resolution of pulmonary thromboembolism (PE) can lead to the development of chronic thromboembolic pulmonary hypertension (CTEPH)^19^, a progressive disease within group 4 of the clinical classification of pulmonary hypertension that is characterized by a poor prognosis^20^. A recent study using a refined computed tomography (CT) imaging method identified that 74% of patients with acute PE had residual thrombi^21^. Furthermore, residual thrombi 1 month after acute PE were independent predictors of residual thrombi at 1 year^21^. Although data in cancer patients are scarce, recurrent VTE is an independent risk factor for developing CTEPH, and attention should be paid to its risk of chronic complications^22–24^. Therefore, to avoid late-stage complications, such as PTS or CTEPH, clot regression in the acute phase of VTE is vital for CAT patients.

Thrombus regression with rivaroxaban was studied in a sub-analysis of the EINSTEIN pulmonary embolism study^25,26^. In this report, CT-evaluated complete clot resolution was observed in 44% of patients with acute PE, and the mean relative decrease in the percentage of vascular obstruction was 59% after 3 weeks of anticoagulant treatment with rivaroxaban^26^. In addition, the J-EINSTEIN study that investigated the efficacy of low-dose rivaroxaban for VTE in Japanese patients demonstrated a complete thrombus resolution rate of 26.7%, 22 days after rivaroxaban administration^27^. These results suggest that rivaroxaban may have a clot regression effect against VTE^28^. However, the clot-regression effect of rivaroxaban in cancer patients with VTE who have hypercoagulation status has not been fully evaluated.

To address this, in this study, we directly investigated the clot regression effect of rivaroxaban, assessed by CT imaging, in CAT patients with high risks of thrombosis and bleeding.

## Methods

### Study population

**Figure 1:**
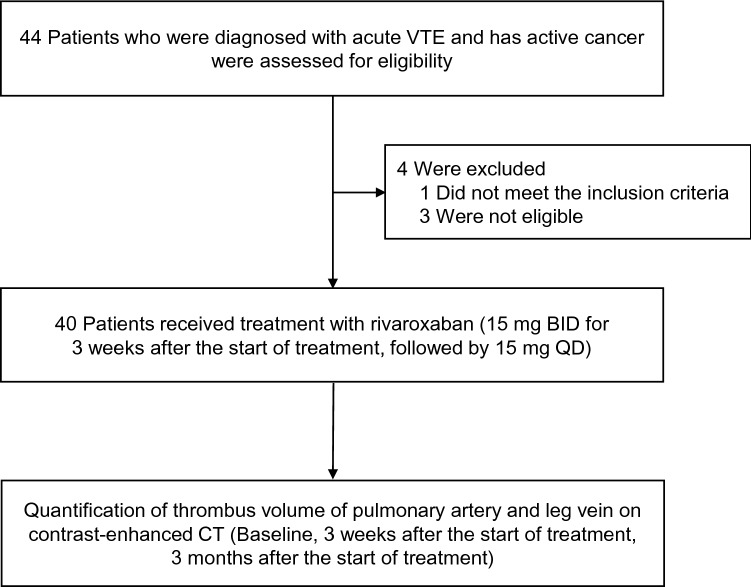
Patient selection flowchart. BID, twice a day; CT, computed tomography; QD, once a day; VTE, venous thromboembolism.

Figure 1 shows the flowchart of patient selection for this single-arm, open-label, prospective interventional study^29^. From June 2018 to June 2021, patients aged 20–75 years with active cancer and acute VTE diagnosis (symptomatic or asymptomatic PE, proximal DVT in the lower or upper extremity, and thrombus in the superior or inferior vena cava) were enrolled. Active cancer was defined as a new, recurrent, or metastatic disease within 180 days prior to obtaining study consent, or the presence of cancer treatment within 180 days. Exclusion criteria were as follows: patients who were treated with anticoagulation or thrombolytic therapies, patients with contraindications to rivaroxaban, distal DVT limited to the lower leg, creatinine clearance rate less than 30 mL/min, life expectancy less than 6 months, patients with serious complications (hepatic dysfunction corresponding to Child–Pugh classification B or C; active hemorrhage, such as intracranial hemorrhage or gastrointestinal hemorrhage; systolic blood pressure ≥ 180 mmHg or diastolic blood pressure ≥ 110 mmHg), and patients who were pregnant or lactating.

This study was approved by the institutional review board of the Kyoto Prefectural University of Medicine (ERB-C-891–2), based on the Helsinki Declaration, and registered in the Japan Registry of Clinical Trials (jRCTs051180077). Written informed consent was obtained from all patients. Date of first enrollment was 18/Aug/2018.

### Treatment protocol and data collection

Eligible patients received the standard treatment of rivaroxaban approved in Japan (15 mg twice a day for 3 weeks after treatment initiation, followed by 15 mg once a day; this protocol regimen is different from the dose approved in other countries). Protocol treatment was continued for 3 months after the initiation of rivaroxaban treatment.

Patient background information and their current clinical status, including vital signs, past medical history, current treatments, types of cancer, laboratory data, pulmonary embolism severity index (PESI) scores for patients with PE, HAS-BLED scores^30,31^, and VTE-BLEED scores^32^ were noted. Creatinine clearance was calculated using the Cockcroft-Gault formula. Data from contrast-enhanced CT examinations were archived at the time of study registration (baseline), 21 ± 4 days after (3 weeks), and 90 ± 14 days after (3 months) the initiation of rivaroxaban treatment. In patients with proximal DVT, contrast-enhanced CT after ultrasound diagnosis was performed to investigate the presence of pulmonary embolism. At the time of scanning, the lower-limb thrombus was concomitantly evaluated. CT data were assembled to quantify thrombus volume.

### Endpoints

The primary endpoint of this study was the clot-regression ratio, evaluated using contrast-enhanced CT imaging. By comparison to the clot volume at baseline, the ratio of clot volume regression was calculated at 3 weeks and 3 months after the initiation of rivaroxaban treatment. Complete thrombus resolution, defined as thrombus disappearance, was also investigated. Secondary endpoints included the recurrence of symptomatic VTE (fatal and non-fatal PE or recurrent DVT) and hemorrhagic complications during the treatment protocol. PE recurrence was defined as a new defect on pulmonary angiography, CT, or lung perfusion scintigraphy with normal ventilation, or a new PE confirmed at autopsy. DVT recurrence was defined as new thrombi, an increase in thrombus diameter of 4 mm or more on echography, or a new contrast defect on contrast-enhanced CT. Regarding hemorrhagic complications, major bleeding was defined using the International Society on Thrombosis and Hemostasis guidelines as fatal bleeding and/or symptomatic bleeding in a critical area or organs (such as intracranial and retroperitoneal regions), and/or bleeding causing a decrease in hemoglobin level of 2 g/mL or more, or leading to transfusion of two or more units of whole blood or red cells^33^. Non-major bleeding was defined as not meeting the aforementioned criteria.

### Quantification of thrombus volume

A method of quantifying the amount of thrombus in CT images is used in general^34^. Unfortunately, we could not use the software to automatically measure the thrombus volume, therefore, thrombus volume was quantified using a Ziostation 2 workstation (version 2.9, Ziosoft Inc., Tokyo, Japan) by manually tracing the clot of VTE within the contrast-enhanced CT image. All pulmonary artery thrombi, proximal lower- or upper-extremity thrombi, and superior or inferior vena cava thrombi were traced to calculate the total thrombus mass volume. If a thrombus could not be detected in the CT image at 3 weeks or 3 months, the thrombus volume was determined to be zero, and complete thrombus resolution was confirmed. Thrombus volume measurements were examined by the independent observer of thrombus evaluation committee for their accuracy. Measurement and evaluation points were both performed in a blinded fashion.

### Statistical analysis

Due to the exploratory nature of this study, the number of cases was determined according to the feasibility of the institution. In the sub-analysis of the EINSTEIN PE study^23^, the clot-regression rate on CT assessment was 59% (standard deviation SD, 37%). As our study was limited to CAT patients with a high thromboembolic risk, the average clot-regression rate was set at 20% (SD, 37%). The required number of patients was 38, which corresponded to a one-sided significance level of 0.025 and a power of 0.9. Therefore, the sample size was set at 40 cases to exceed this required number and provide for exclusions. A full analysis set (FAS) was used to assess efficacy, and a safety analysis set (SAS) was used for adverse events (AEs). The FAS was applied to all patients who received the protocol treatment, with the exclusion of those with post-enrollment ineligibility and those with unobtainable information after enrolment. The SAS was applied to all patients who received at least one dose of treatment.

The clot-regression ratio was defined as the ratio of the regressed thrombus volume at 3 weeks and 3 months post rivaroxaban initiation to the thrombus volume at baseline, both expressed as a percentage. The mean and 95% confidence intervals (CIs) for the whole population were calculated according to the t-distribution. If either of the 95% CI values did not contain 0%, thrombus regression was considered statistically significant (identical to the paired t-test for each clot-regression rate). This corresponds to a test with a one-sided significance level of 0.025 if the null hypothesis is defined as a clot-regression rate of 0%. For the secondary endpoints, the frequency and percentage of symptomatic VTE recurrence and hemorrhagic complications were calculated during the study period. The incidence and severity of all AEs for the SAS were also collected during the study period.

Variables are reported as mean ± SD or 95% CI. The corresponding variables were compared using paired t-tests. Data were analyzed using the unpaired Student’s t-test for comparisons between two independent groups or one-way analysis of variance for multiple comparisons. Categorical variables were compared using the Chi-squared test. Statistical significance was set at *p* < 0.05. All statistical analyses were performed using SPSS Statistics software (version 22 IBM Corp., NY, USA) and Statistical Analysis System (SAS® version 9.4 SAS Institute, Cary, NC).

## Results

### Baseline characteristics in CAT patients

After an initial screening of 44 CAT patients, 40 were enrolled in this study. The clinical characteristics of the patients are summarized in Table 1. The patients (55% male) had a mean age of 61.9 ± 10.2 years and a mean body mass index (BMI) of 23.8 ± 5.1. Most patients (72.5%) had PE complications. Among patients with PE, the mean PESI score was 107 ± 21.2, indicating a high risk of 30-day mortality. Approximately a quarter (27.5%) of the patients had only proximal DVT without PE. Although 30% of patients had liver dysfunction, renal function was adequately conserved. No patients received antiplatelet therapy or had thrombophilia. The mean HAS-BLED score at enrolment was 1.2 ± 0.9, and VTE-BLEED score was 4.55 ± 1.40. Regarding cancer-type, 22.5% of patients had genitourinary cancer and 45% of patients had gastrointestinal cancer, both of which have a high bleeding risk with DOACs therapy.

**Table 1:** Clinical characteristics of patients with VTE.

Characteristics	Rivaroxaban (N = 40)
Age, years	61.9 ± 10.2
Male, n (%)	22 (55)
Weight, kg	62.8 ± 14.6
BMI	23.8 ± 5.1
**Venous thromboembolism, n (%)**	
DVT only	11 (27.5)
PE	29 (72.5)
PESI score	107 ± 21.2
D-dimer, μg/mL	15.7 ± 10.5
**Cancer type, n (%)**	
Breast	4 (10)
Upper gastrointestinal	12 (30)
Lower gastrointestinal	6 (15)
Lung	2 (5)
Genitourinary	9 (22.5)
Brain	2 (5)
Blood	2 (5)
Skin	1 (2.5)
Others	3 (7.5)
Liver dysfunction, n (%)	12 (30)
Renal dysfunction, n (%)	1 (2.5)
**Creatinine clearance, n (%)**	
> 80 mL/min	20 (50)
50–80 mL/min	18 (45)
30–50 mL/min	2 (5)
< 30 mL/min	0 (0)
Antiplatelet therapy, n (%)	0 (0)
Known thrombophilia, n (%)	0 (0)
HAS-BLED score	1.2 ± 0.9
VTE-BLEED score	4.55 ± 1.40

Data are presented as number (%) or mean ± standard deviation.

*BMI* Body mass index; *DVT* Deep vein thrombosis; *PESI* Pulmonary embolism severity index *PE* Pulmonary thromboembolism; *VTE* Venous thromboembolism.

### Clot regression effect of rivaroxaban in CAT patients

**Figure 2:**
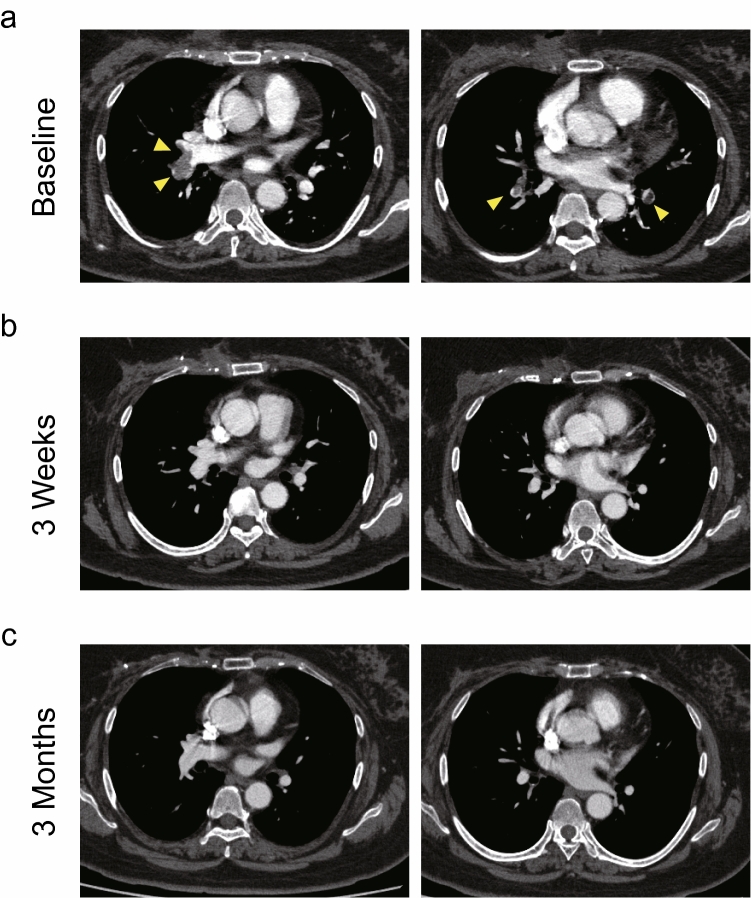
Representative CT scan images of pulomonry thromboembolism in patients with breast cancer who received rivaroxaban treatment. (**a**) Bilateral pulmonary thromboembolism were ditected by contrast-enhanced CT scan at baseline. Yellow arrows show thrombi in pulmonary artery. (**b**) After the treatment with rivaroxaban 30 mg for 3 weeks, pulmonary thrombi were disappeared, achieving complete thrombus resolution. (**c**) There were no venous thromboembolism reccurence after the treatment with rivaroxaban 15 mg for 3 months.

**Figure 3:**
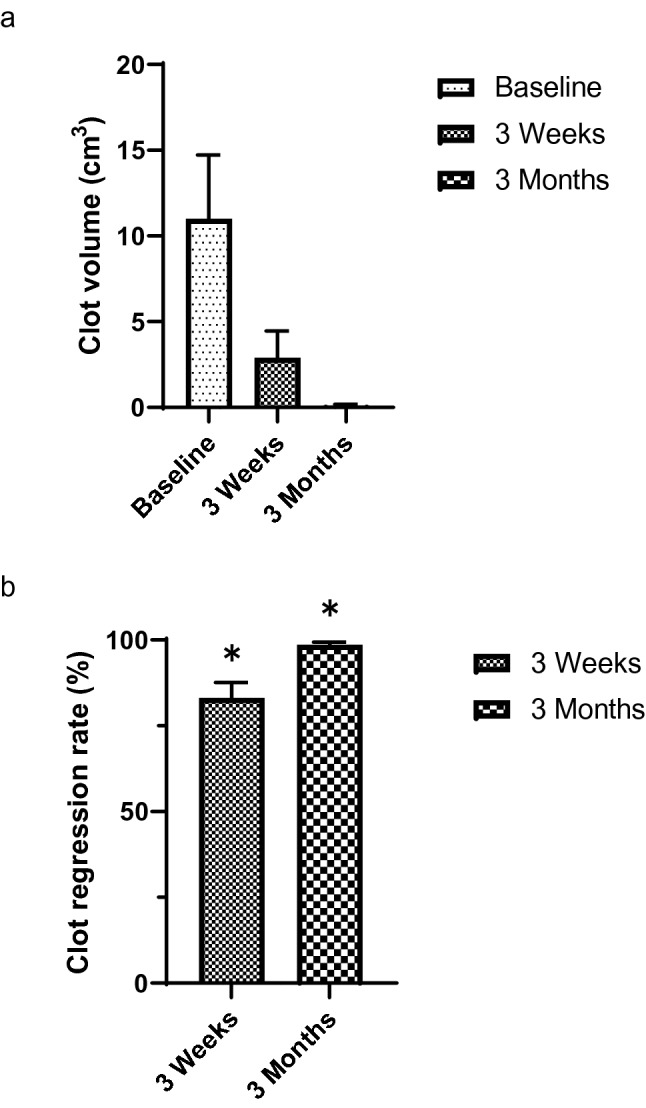
Clot-regression effect of rivaroxaban. (**a**) Clot volume assessed by contrast-enhanced CT at baseline, 3 weeks, and 3 months after rivaroxaban initiation. (**b**) Clot-regression rates 3 weeks and 3 months after rivaroxaban initiation. Data are expressed as mean ± standard error. * *p* < 0.05.

Table 2 shows clot volume and clot-regression rate assessed by contrast-enhanced CT at baseline, 3 weeks, and 3 months after rivaroxaban treatment (Fig. 2). Total clot volume was decreased after 3 weeks (2.89 cm^3^; range, 0–54.62 cm^3^) and 3 months (0.11 cm^3^; range, 0–1.38 cm^3^) of rivaroxaban treatment compared with baseline (11.00 cm^3^; range, 0.05–142.25 cm^3^) (Fig. 3a). The clot-regression effect seemed to continue even after the rivaroxaban dose was reduced to 15 mg. The primary endpoint, the clot-regression rate, was calculated as 83.1% (95% CI, 73.9–92.3%) at 3 weeks and 98.7% (95% CI, 97.1–100.0%) at 3 months, respectively (Fig. 3b), indicating statistically significant clot regression at both 3 weeks and 3 months after rivaroxaban treatment. In sensitivity analysis considering dropouts that had clot-regression rates of zero, significant differences were still observed with clot-regression rates of 74.7% (95% CI, 63.2–86.3%) and 64.1% (95% CI, 48.9–79.4%) at 3 weeks and 3 months, respectively. In addition, complete thrombus resolution was achieved in 36.1% and 80.8% of patients at 3 weeks and 3 months, respectively.

**Table 2:** Clot-regression effect of rivaroxaban.

	Baseline (N = 40)	3 weeks (N = 36)	3 months (N = 26)
Clot volume, cm^3^ [range]	11.00 [0.05–142.25]	2.89 [0–54.62]	0.11 [0–1.38]
Clot-regression rate, % (95% CI)	–	83.1 (73.9–92.3)*	98.7 (97.1–100.0)†
Complete resolution, n (%)	–	13 (36.1)	21 (80.8)

Clot volume, clot-regression rate and complete resolution are presented as mean (range), mean (95% CI) and number (%), respectively. CI: Confidence interval. The clot-regression rate is the ratio of clot volume regression after 3 weeks or 3 months to the clot volume at baseline. *74.7 (95% CI: 63.2–86.3) in sensitivity analysis with 4 dropouts considered to have a clot-regression rate of 0. †64.1 (95% CI: 48.9–79.4) in sensitivity analysis with 14 dropouts considered to have a clot-regression rate of 0. CI, confidence interval.

### Clinical efficacy and safety of rivaroxaban in CAT patients

We assessed the recurrence of symptomatic VTE and hemorrhagic complications as secondary endpoints (Table 3). Among the 40 patients who received protocol treatment, only one patient had recurrent VTE (recurrent DVT) after dose reduction of rivaroxaban. Regarding safety concerns, hemorrhagic complications occurred in 12.5% of patients at 3 weeks and 15.2% at 3 months (*p* = 0.50). In total, seven patients had major bleeding; however, there was no fatal bleeding during the 3-month study period. Although gastrointestinal bleeding was most commonly observed, there was no significant difference between the intensive high-dose period of rivaroxaban and the low-dose period (5.0% vs. 6.0%, *p* = 0.61). One patient experienced traumatic intracranial hemorrhage. Furthermore, the administration of intensive high-dose rivaroxaban for 3 weeks did not increase the non-major bleeding rate compared to that with low-dose rivaroxaban for 3 months (2.5% vs. 9.0%, *p* = 0.23).

**Table 3:** Clinical efficacy and safety.

	3 weeks (N = 40)	3 months (N = 33)	*p* value
**Efficacy**			
Recurrent VTE, n (%)	0	1 (3.0)	0.45
**Type of recurrent VTE, n (%)**			
Fatal PE	0	0	1.00
Nonfatal PE	0	0	1.00
Recurrent DVT	0	1 (3.0)	0.45
**Safety**			
Hemorrhagic complications, n (%)	5 (12.5)	5 (15.2)	0.50
Major bleeding, n (%)	4 (10)	3 (9.0)	0.60
Fatal	0	0	1.00
Nonfatal	4 (10)	3 (9.0)	0.60
Traumatic intracranial hemorrhage	0	1 (3.0)	0.45
Gastrointestinal bleeding	2 (5.0)	2 (6.0)	0.61
Intraabdominal hemorrhage	1 (2.5)	0	0.54
Hypermenorrhea	1 (2.5)	0	0.54
Non-major bleeding, n (%)	1 (2.5)	3 (9.0)	0.23
Epistaxis	1 (2.5)	1 (3.0)	0.70
Gastrointestinal bleeding	0	2 (6.0)	0.20

Data are presented as numbers (%). DVT, deep vein thrombosis; PE, pulmonary thromboembolism; VTE, venous thromboembolism.

Fourteen patients (35%) experienced AEs during rivaroxaban treatment (Table 4). Seven patients (17.5%) had serious AEs, including one patient with gastrointestinal bleeding and one patient with severe anemia without obvious bleeding. Among the 40 CAT patients treated with rivaroxaban, 15 patients (37.5%) prematurely discontinued treatment. Ten patients (25%) discontinued treatment early due to AEs, including hemorrhagic complications, and two patients (5%) died due to cancer progression.

**Table 4:** Adverse events.

	Rivaroxaban (N = 40)
**Adverse event, n (%)**	
Any event during treatment	14 (35)
Any serious event during treatment	7 (17.5)
Liver dysfunction	2 (5)
Gastrointestinal bleeding	1 (2.5)
Severe anemia	1 (2.5)
Ileus	1 (2.5)
Contrast allergy	1 (2.5)
Pseudoaneurysm	1 (2.5)
Premature discontinuation of treatment, n (%)	15 (37.5)
Adverse event	10 (25)
Overdose	1 (2.5)
Death	2 (5)
Others	2 (5)

Data are presented as numbers (%).

### Subgroup analysis for the clot regression effect and safety of rivaroxaban in CAT patients

**Figure 4:**
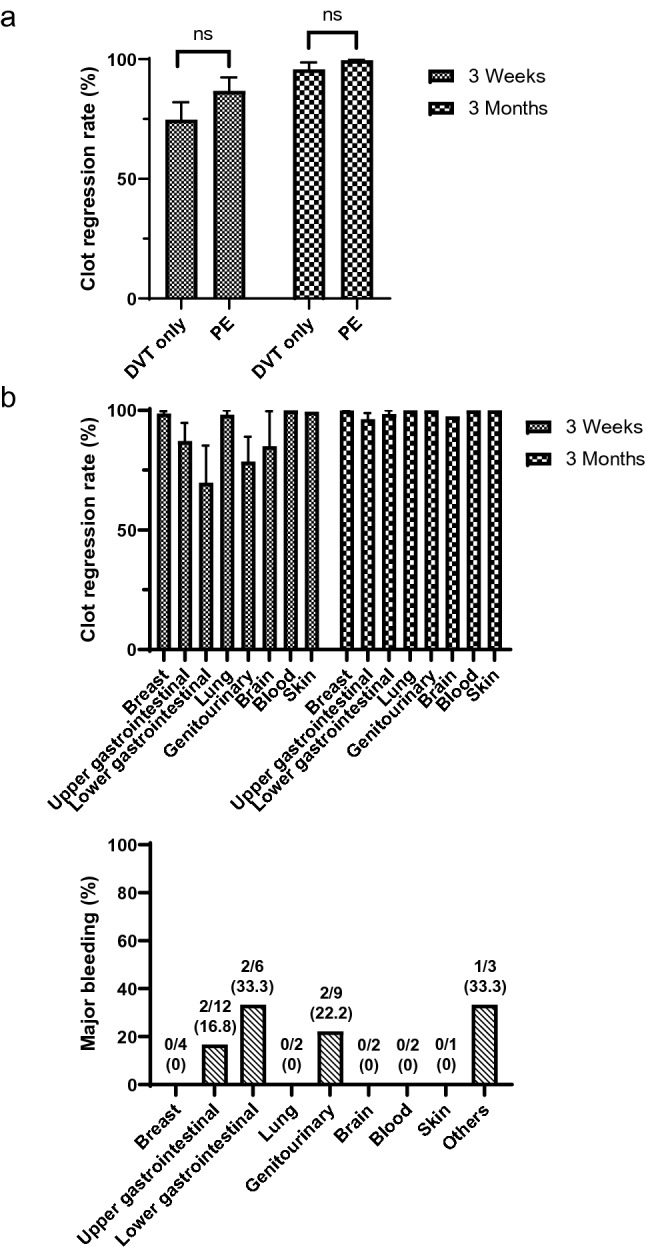
Clot-regression rate and major bleeding according to the PE involvement and cancer type. (**a**) Clot-regression rate between DVT-only patients and PE patients. (**b**) Clot-regression rate and major bleeding based on the cancer type. Data are expressed as mean ± standard error or percentage. ns, not significant; DVT, deep vein thrombosis; PE, pulmonary thromboembolism.

We explored the clot-regression effect of rivaroxaban and major bleeding in subgroups of CAT patients. First, we investigated the difference in clot-regression rate between DVT-only patients and PE patients. There were no significant differences in the clot-regression effect between two groups at 3 weeks (74.7 ± 24.2%, DVT only; 86.7 ± 28.2%, PE; *p* = 0.22) and 3 months (95.7 ± 7.1%, DVT; 99.6 ± 1.5%, PE; *p* = 0.24) (Fig. 4a). Although statistical analysis could not be performed due to the small sample size, sufficient thrombus regressions were obtained in almost all cancer types (Fig. 4b, top). Regarding hemorrhagic complications, major bleedings were frequently observed in patients with gastrointestinal cancers and genitourinary cancer (Fig. 4b, bottom), similar to results in previous reports^15^.

**Figure 5:**
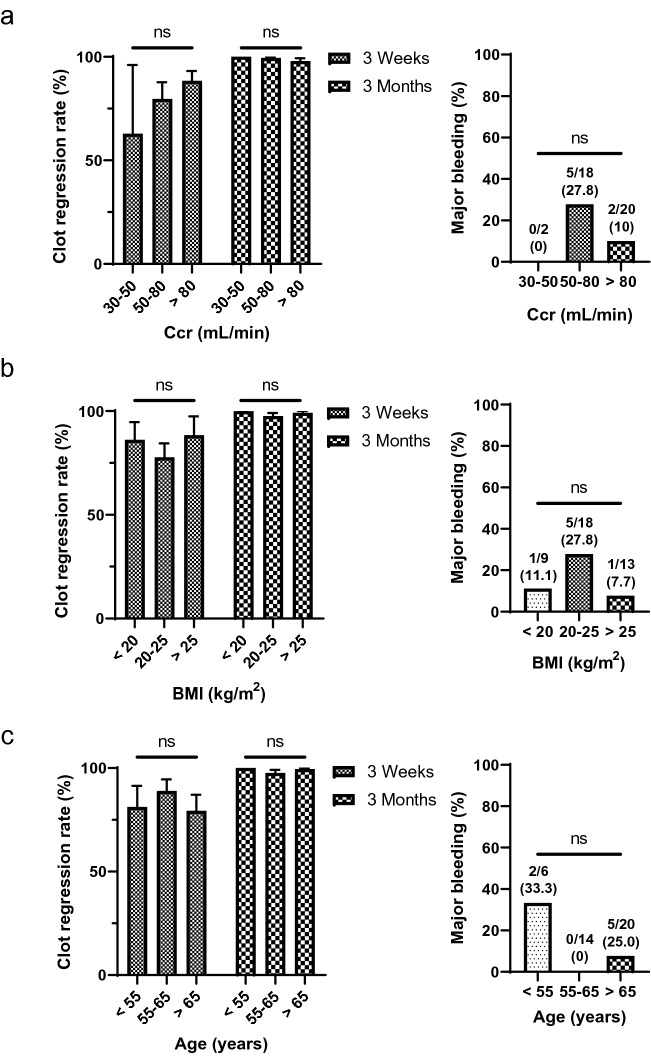
Subgroup analysis for the clot regression effect and safety of rivaroxaban in CAT patients. (**a**) Clot-regression rate and major bleeding based on creatine clearance. (**b**) Clot-regression rate and major bleeding based on body mass index. (**c**) Clot-regression rate and major bleeding based on age. Data are expressed as mean ± standard error or percentage. BMI, body mass index; Ccr, creatine clearance; ns, not significant.

Figure 5 shows subgroup analysis according to creatinine clearance, BMI, and age. Clot-regression rate did not depend on creatinine clearance (3 weeks, *p* = 0.37; and 3 months, *p* = 0.62), BMI (3 weeks, *p* = 0.57; and 3 months, *p* = 0.43), and age (3 weeks, *p* = 0.72; and 3 months, *p* = 0.45). Previously, renal dysfunction and elderly age were considered risk factors for bleeding^35^. However, major bleeding was not significantly increased in these high-risk groups in our study (creatinine clearance, *p* = 0.28; and age, *p* = 0.05).

## Discussion

In this study, we explored the clot regression effects of rivaroxaban in CAT patients. While current guidelines recommend LMWH for the treatment of CAT patients^10,11^, DOACs have been recently reported to be non-inferior to LMWH in treating VTE recurrence and major bleeding in CAT patients^12,13,15^. Consequently, DOACs can be considered an alternative to LMWH for the treatment of CAT patients, with caution against bleeding, particularly in gastrointestinal and genitourinary cancers. Although the usefulness of DOACs in VTE recurrence has been investigated^12,13^, its clot regression effect has not been fully explored, particularly in cancer patients with an increased risk of thrombosis and hemorrhage. Thrombus volume reduction and clot resolution in the acute phase of VTE can prevent the development of late-stage complications, such as PTS and CTEPH^17–19^. Here, we showed that rivaroxaban significantly decreased clot volume and had a clot-regression effect in CAT patients with acceptable hemorrhagic complications.

VTE is a common complication in patients with cancer and a serious health concern, associated with poor prognosis and reduced quality of life^36^. In cancer patients, a hypercoagulation state could result directly from the aberrant expression of tissue factors by tumor cells, or indirectly by leukocytes or endothelial cells, leading to thrombosis^37^. Anticoagulation therapy in cancer patients poses risks for both thrombosis and bleeding^5^, stressing the need for careful examination of the risk–benefit relationship^38^. Compared with LMWH in CAT patients, rivaroxaban had a lower VTE recurrence rate, comparable major bleeding risk, and a higher clinically relevant non-major bleeding rate^15^. In another study, rivaroxaban had a significantly lower VTE recurrence rate and an equal risk of bleeding compared to LMWH in the treatment of CAT patients^39^. A sub-analysis of the EINSTEIN pulmonary embolism study revealed that complete clot resolution occurred in 44% of patients after 3 weeks of rivaroxaban treatment for VTE^26^. The J-EINSTEIN study that evaluated low-dose rivaroxaban in Japanese VTE patients reported thrombus normalization in 30.8% of patients after 3 weeks of rivaroxaban 15 mg bid, and 62.0% at the end of the intended treatment period^27^. These studies were not limited to cancer patients and included only a small number of CAT patients. Cancer patients are in a hypercoagulable state^37^, and the clot regression effect of rivaroxaban may be decreased in these patients. However, we observed adequate clot-regression rates of 83.1% and 98.7% at 3 weeks and 3 months, respectively, after rivaroxaban initiation. Furthermore, complete clot resolution was achieved in 36.1% of patients 3 weeks after rivaroxaban initiation and 80.8% of patients after 3 months. Although there is a difference in the evaluation method of thrombi, our results seem to be comparable to EINSTEIN and J-EINSTEIN study. Consistent with previous reports^26,27^, our study demonstrated that rivaroxaban has a sufficient clot regression effect even in cancer patients with a hypercoagulation state. Treatment with rivaroxaban for 3 months can result in a marked regression or disappearance of thrombi in most CAT patients.

Hemorrhagic complications are common during rivaroxaban therapy in CAT patients and can be fatal. In the SELECT-D study^15^, the major bleeding rate at 6 months was 6% for rivaroxaban, which was comparable to that of dalteparin in the treatment of CAT patients. Gastrointestinal cancer is an independent risk factor for major bleeding during anticoagulation treatment^35^. In the EPIPHANY study^40^, a registry study of PE patients with cancer, 92% of patients were treated with LMWH, and 5.0% had major bleeding within 15 days. Patients in our study had a 17.5% rate of major bleeding complications during 3 months treatment with rivaroxaban, mainly gastrointestinal bleeding. One patient experienced traumatic intracranial hemorrhage. However, initial intensive high-dose treatment with rivaroxaban did not increase hemorrhagic complications. In light of this observation, rivaroxaban therapy for the treatment of VTE in patients with cancer should be carefully monitored for bleeding complications.

Patients aged greater than 65 years or with a creatinine clearance of less than 50 mL/min have been reported to have a high bleeding risk during anticoagulation treatment for VTE^41^. Patients aged greater than or equal to 75 years who received rivaroxaban had a higher bleeding rate than younger patients in the ROCKET AF trial^42^. Therefore, we excluded patients aged greater than or equal to 75 years in this study due to safety concerns. In the subgroup analysis, major bleeding was not significantly increased in patients with creatinine clearance rates of lower than 50 mL/min or those within 65–75 years of age. Although our study excluded patients aged over 75 years or patients with creatinine clearance rate less than 30 mL/min who has high bleeding risk, our findings suggest that rivaroxaban can be safely used in patients with moderate bleeding risk.

Oral rivaroxaban can be used to treat VTE using a single-drug approach. The HoT-PE study which included 6.2% of patients with active cancer demonstrated the efficacy and safety of rivaroxaban for early discharge and home treatment in low-risk PE patients^43^. EPIPHANY study^40^ demonstrated the outcomes and complications in cancer-associated PE. In EPIPHANY study, 92.2% of patients were initially treated with LMWH, and recurrence of PE at 15 days was observed in 4.0%. Although our PE patients had a high PESI score of 107 (categorized as high-risk), there was no PE recurrence during the treatment period with rivaroxaban. Moreover, rivaroxaban decreased clot volume by as much as 98.7%, and 80.8% of patients showed complete resolution in our study. To our knowledge, this is the first report to directly evaluate the clot-regression rate of rivaroxaban in CAT patients. Even in CAT patients with hypercoagulability, rivaroxaban sufficiently reduced the clot volume and thus may prevent late-stage complications. Intensive high-dose rivaroxaban for 3 weeks did not increase the risk of hemorrhagic complications. Our findings also suggest that rivaroxaban may be effective in patients with large thrombus volumes.

This study has some limitations. The first limitation is that it was a single-arm, open-label study with a small sample size. Detailed subgroup analysis or examination of potential factors affecting clot regression rate and the relationship with the clinical course were not possible due to the small number of cases. Though it aimed to explore the clot regression effect of rivaroxaban in CAT patients, clot regression occurs both due to rivaroxaban and the physiological thrombolytic function of the body. There was no control group in this study to neutralize the effect of natural thrombolysis. However, the rate of natural thrombolysis being small, this study can be presumed to depict the clot regression rate of rivaroxaban using a time-course analysis. Additionally, we did not compare the clot regression effect and safety of rivaroxaban with those of other anticoagulants. The difference of approved dose of rivaroxaban between Japan and other countries might affect the clot regression rate, and we could not directly compare the clot regression ratio with studies at other doses. Furthermore, it was not possible to examine the clot regression effect according to cancer type. Mucinous carcinomas, with frequently-produced mucin, develop more VTE compared to non-mucinous carcinomas^44^. Therefore, a cancer-type dependent difference in the clot-regression effect might exist and should be evaluated for rivaroxaban in the future. Despite these limitations, this study provides new insights into the clot regression effect of rivaroxaban in CAT patients.

In summary, this study revealed that rivaroxaban had a sufficient clot-regression effect for the treatment of VTE in cancer patients. However, clinical use of rivaroxaban in patients with cancers should be met with caution of bleeding complication.

## Data Availability

The datasets used and/or analyzed during the current study are available from the corresponding author on reasonable request.
